# Single-Cell Profiling of Tumor Microenvironment Heterogeneity in Osteosarcoma Identifies a Highly Invasive Subcluster for Predicting Prognosis

**DOI:** 10.3389/fonc.2022.732862

**Published:** 2022-04-06

**Authors:** Junfeng Guo, Hong Tang, Pan Huang, Junfeng Guo, Youxing Shi, Chengsong Yuan, Taotao Liang, Kanglai Tang

**Affiliations:** ^1^ Department of Orthopaedics/Sports Medicine Center, State Key Laboratory of Trauma, Burn and Combined Injury, Southwest Hospital, Third Military Medical University, Chongqing, China; ^2^ Department of Stomatology, The 970th Hospital of the Joint Logistics Support Force, Yantai, China

**Keywords:** osteosarcoma, metastasis, scRNA-seq, tumor microenvironment, prognosis

## Abstract

Osteosarcoma is the most common malignant bone tumor in adolescents, and metastasis is the key reason for treatment failure and poor prognosis. Once metastasis occurs, the 5-year survival rate is only approximately 20%, and assessing and predicting the risk of osteosarcoma metastasis are still difficult tasks. In this study, cellular communication between tumor cells and nontumor cells was identified through comprehensive analysis of osteosarcoma single-cell RNA sequencing (scRNA-seq) and bulk RNA-seq data, illustrating the complex regulatory network in the osteosarcoma microenvironment. In line with the heterogeneity of osteosarcoma, we found subpopulations of osteosarcoma cells that highly expressed COL6A1, COL6A3 and MIF and were closely associated with lung metastasis. Then, BCDEG, a reliable risk regression model that could accurately assess the metastasis risk and prognosis of patients, was established, providing a new strategy for the diagnosis and treatment of osteosarcoma.

## Introduction

Osteosarcoma (OS) is the most common primary malignant bone tumor, accounting for approximately 60% of all pediatric malignancies, and is the second leading cause of cancer-related death in adolescents (1–4). Seper et al. reported that the first confirmed finding of OS occurred in a dinosaur (a specimen of *Centrosaurus apertus*), dating from approximately 77.0–75.5 million years ago (5). Experts have noted that OS mainly occurs in the proximal tibia, proximal humerus, and distal femur and that its clinical manifestations mainly include local pain, swelling, and limited joint mobility (6, 7). For OS, local therapy alone is insufficient, as 80-90% of all patients with seemingly localized disease will develop metastasis (6).

Tumor metastasis is the main cause of treatment failure and death in patients with OS. Metastasis to the lungs is the most common type and has a great impact on the prognosis of patients (8). Because of the extensive application of neoadjuvant chemotherapy, the prognosis of patients with localized OS has been significantly improved, with a 5-year survival rate reaching 60-70%. However, the survival rate of those with pulmonary metastasis is still poor, at only 20-30% (9, 10). This situation highlights the urgency of studying the biological behavior and genetic characteristics of OS metastasis. An accurate description of the process of tumor metastasis will help to enhance our understanding of OS and improve clinical treatment effects and patient survival (11, 12). In previous studies, researchers have utilized a variety of assessment methods to investigate OS. Sheen et al. used two radiomics features to develop a logistic regression imaging model of OS metastasis (13), but its sensitivity was slightly low, which probably led to delayed treatment. Flores et al. assessed the blood‐based biomarkers for prognostication in OS patients (14); however, circulating biomarkers are unstable and susceptible. Tissue biopsy is of great necessity in the diagnosis of OS, and this approach is more reliable than other methods for predicting OS progression and metastasis.

The combined application of bone scintigraphy and CT is often used to identify metastases of OS (15). However, imagological examination alone is insufficient, and pulmonary micrometastases are often undiagnosed, leading to poor prognosis (16). Considering the deficiencies in early pulmonary metastasis diagnosis and resulting poor survival, there is an urgent need for pulmonary metastatic biomarkers for OS. Single-cell RNA sequencing (scRNA-seq), through the analysis of transcripts within an individual cell, can distinguish tumor cells from nontumor cells and allow exploration of the interrelationships between cells in the tumor microenvironment. Breakthroughs have been made in the study of tumor cell heterogeneity, proliferation and metastasis (17, 18). OS is highly heterogeneous and has many cell subpopulations with different biological functions that evolve during tumor progression (19). scRNA-seq of OS cells is conducive to identifying new cell types, exploring tumor heterogeneity and revealing different developmental trajectories (20), which can provide new theoretical support for further research on the molecular mechanisms of OS progression and metastasis.

## Materials and Methods

### Data Sources and Processing

Single-cell sequencing data and clinical information for 11 OS patients were downloaded from GSE152048. The 10X genomics data for each individual sample were loaded by the Seurat package (v4.0.0) in R software (v4.0.2) (21). Low-quality cells with ≤ 300 detected genes or ≥ 10% mitochondrial genes were removed. Feature counts for each cell were divided by the total and multiplied by 10,000. The log(x+1) method was used to perform natural-log transformation. The top 2,000 highly variable genes (HVGs) in the normalized expression matrix were identified, centered, and scaled before we performed principal component analysis (PCA) based on these HVGs. Batch effects were removed with the Harmony package (v1.0) of R based on the top 50 PCA components identified (22).

Eighteen tumor-normal pairs of osteosarcoma RNA-sequencing data were downloaded from GSE99671. The edgeR package (v 3.30.3) was used to identify up- and down-regulated genes in osteosarcoma (log2FoldChange >1 and adjusted P-value < 0.1). Bulk RNA-seq and clinical data for 80 OS patients from the TARGET database and 53 from GSE21257 were collected. Patients from TARGET were randomly divided into two groups: a training group (40), and an internal verification group (40). The 53 patients from GSE21257 were used as an external training group. All the raw data are available in the GEO database (https://www.ncbi.nlm.nih.gov/geo/).

### Identification of Cell Types

Based on harmony-corrected data, k-nearest neighbors were calculated, and a shared nearest neighbor (SNN) graph was constructed. Then, the modular function was optimized to realize cluster recognition based on the clustering algorithm. The identified clusters were visualized on a 2D map by employing the uniform manifold approximation and projection (UMAP) dimensional reduction technique. We first used the “SingleR” package (R software) as an auxiliary tool to identify cells, and then used the Wilcoxon rank-sum test with Bonferroni correction to identify marker genes for double annotation of cell clusters.

### Cell-Cell Communication

Crosstalk between OS cells and other cells within the tumor microenvironment was calculated with the iTALK package (v0.1.0) in R (23). The top 50% of highly expressed genes were uploaded to the ligand-receptor database to determine their locations within an intercellular signaling network.

### Gene Set Enrichment Analysis

Gene set enrichment analysis (GSEA) was used to detect whether the PI3K-AKT signaling pathway was significantly enriched in the subpopulations of OS cells. The enrichment score (ES) and statistical significance (nominal P value) of the ES were calculated; a positive ES suggested that PI3K-AKT signaling was activated in a subpopulation, whereas a negative ES suggested that PI3K-AKT signaling was not activated.

### Pseudotemporal Ordering of Osteosarcoma Cells

Monocle (v2.16.0) aims to resolve cellular transitions during differentiation through pseudotemporal profiling of scRNA-seq data (24). After inputting the cell-gene matrix into the “newCellDataSet” function with its clustering information, it was computed into a lower dimensional space based on the discriminative dimensionality reduction with trees (DDRTree) method, a more recent manifold learning algorithm, and then OS cells were ordered according to pseudotime.

### Generation of the Risk Regression Model

To establish a generalized linear model and minimize the risk of overfitting, we applied the least absolute shrinkage and selection operator (LASSO) penalty in the COX proportional hazards regression model (“glmnet” package, R software). In the modelling process, “lambda” is the variable and causes the gene coefficients to converge. As lambda increases, the coefficients for individual genes gradually approach zero. When the coefficient becomes zero, it means that the expression of the gene no longer affects the risk score and the gene can be removed from the model. We used 10-fold cross-validation to calculate partial-likelihood, a key metric for evaluating model performance. Value of lambda that gives minimum partial-likelihood is optimal, at which the model constructed of candidate genes with nonzero regression coefficients has the best performance. Risk scores for each patient were calculated using the following formula:


$$RS=\Sigma_{i=1}^{N}(e_{i}*c_{i}),$$

where *RS* is the risk score for OS patients; *N* is the number of genes in the model; and the *i*th gene expression and coefficient are denoted by *e*_i_ and *c*_i_ respectively. The independent variables are the expression of the modelling genes for each patient, and the dependent variable is the risk score.

According to the median scores for each group, the patients were divided into high-risk and low-risk subgroups.

### Cell Culture

The human OS cell line HOS was kindly provided by Procell Life Science & Technology Co., Ltd (Wuhan, China) and cultured in Dulbecco’s Modified Eagle Medium/Nutrient Mixture F-12 (DMEM/F-12; Gibco, Grand Island, NY, USA) supplemented with 10% fetal bovine serum (FBS; HyClone, South Logan, UT, USA) and 1% penicillin-streptomycin (Gibco, Grand Island, NY, USA) at 37°C with 5% CO_2_.

### SiRNA Transfect Cells

OS cells were grown to 60-80% confluence. In all, 30 pmol EFEMP2 siRNA, GALNT14 siRNA or negative control siRNA (GenePharma, Shanghai, China) was diluted to 150 µl Opti-MEM^®^ Reduced-Serum Medium (Gibco, Grand Island, NY, USA) respectively and mixed with the diluted Lipofectamine^®^ RNAiMAX Reagent (1:1 ratio; Thermo Fisher Scientific, Waltham, MA, USA). The siRNA-lipid complex was incubated for 5 minutes at room temperature and then added to the cells.

### Quantitative RT-PCR

Total RNA was extracted using the SimplyP RNA Kit (Bioer, Hangzhou, China), and then reversed transcription was carried out using the PrimeScript RT reagent Kit (TaKaRa, Tokyo, Japan). Quantitative RT-PCR was performed with SsoAdvanced Universal SYBR Green Supermix reagent (Bio-Rad, Hercules, CA, USA) on a PCR system (Bio-Rad, Hercules, CA, USA) per the manufacturer’s instructions. The primers were designed and synthesized by Sangon Biotech (Shanghai, China), and the sense and antisense primers are listed in **Table S2**.

### Wound Healing

HOS cells (5x10^5^) were seeded in 6-well plates and transfected by siRNA described above. When the cells reached 90-95% confluence as a monolayer, a scratch wound was made as a marked line in the cultures using a sterile pipette tip. Wells were washed three times with phosphate-buffered saline (PBS; 0.01 M) to remove loose or dead cells, and fresh serum-free medium was added. The cells were photographed at 0-, 12- and 24-hour time points after treatment. The wound area was quantitated using ImageJ software (25).

### Statistical Analysis

Bilateral tests were performed for statistical tests. A P value less than 0.05 was considered statistically significant. R software version 4.0.0 (https://www.r-project.org/) was used for analysis. Some R packages, including “dplyr”, “Seurat”, “cowplot”, “Matrix”, “ggplot2”, “reshape2”, “iTALK”, “monocle”, “harmony”, and “survival”, were used in this study.

## Results

### Cell Type Identification in the Osteosarcoma Microenvironment

**Figure 1:**
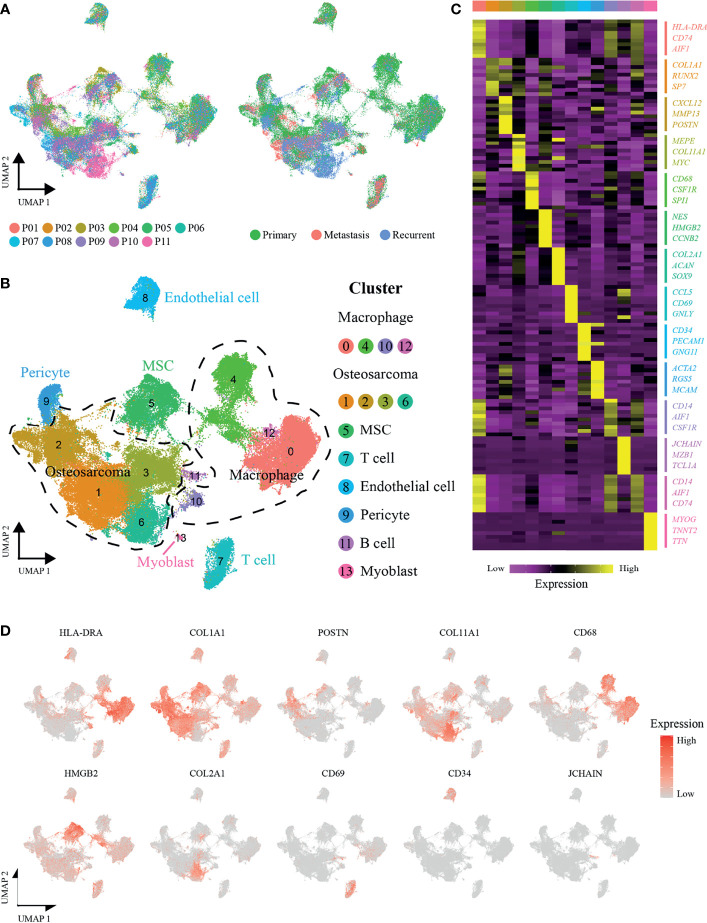
scRNA-seq profiling of OS tumor microenvironments. **(A)** Uniform manifold approximation and projection (UMAP) visualization of cells clustered and color-coded according to the patient (left panel) or disease state (right panel). **(B)** UMAP plot showing annotated and color-coded cell types in the OS microenvironment. **(C)** Heatmap showing the expression of marker genes in the indicated cell types. The top bar color indicates the specific cell type cluster. **(D)** UMAP plot showing expression levels of marker genes for specific cell types.

A total of 11 OS patients with scRNA-seq data were involved in this study (seven primary patients: P01-07, two recurrent patients: P08-09, and two lung-metastatic patients: P10-11) (**Figure 1A**). After quality control and removal of the batch effect between samples, we used the UMAP technique to reduce dimensionality, and graphed the output on a 2D scatter plot (**Figure 1B**). A total of 109,415 single cells were unbiasedly clustered into 14 major identities, and cluster-specific markers were used to annotate cell types: OS cells (COL1A1, CXCL12, MEPE and COL2A1, including osteoblastic OS cells and chondroblastic OS cells); macrophages (HLA-DRA, CD68 and CD14); mesenchymal stem cells (MSCs) (NES, HMGB2 and CCNB2); T cells (CCL5 and CD69); endothelial cells (CD34 and PECAM1); pericytes (ACTA2 and RGS5); B cells (JCHAIN and MZB1) and myoblasts (MYOG) (**Figures 1B–D**). Detailed annotation information and references are given in the **Table S1**.

### Cellular Communication

**Figure 2:**
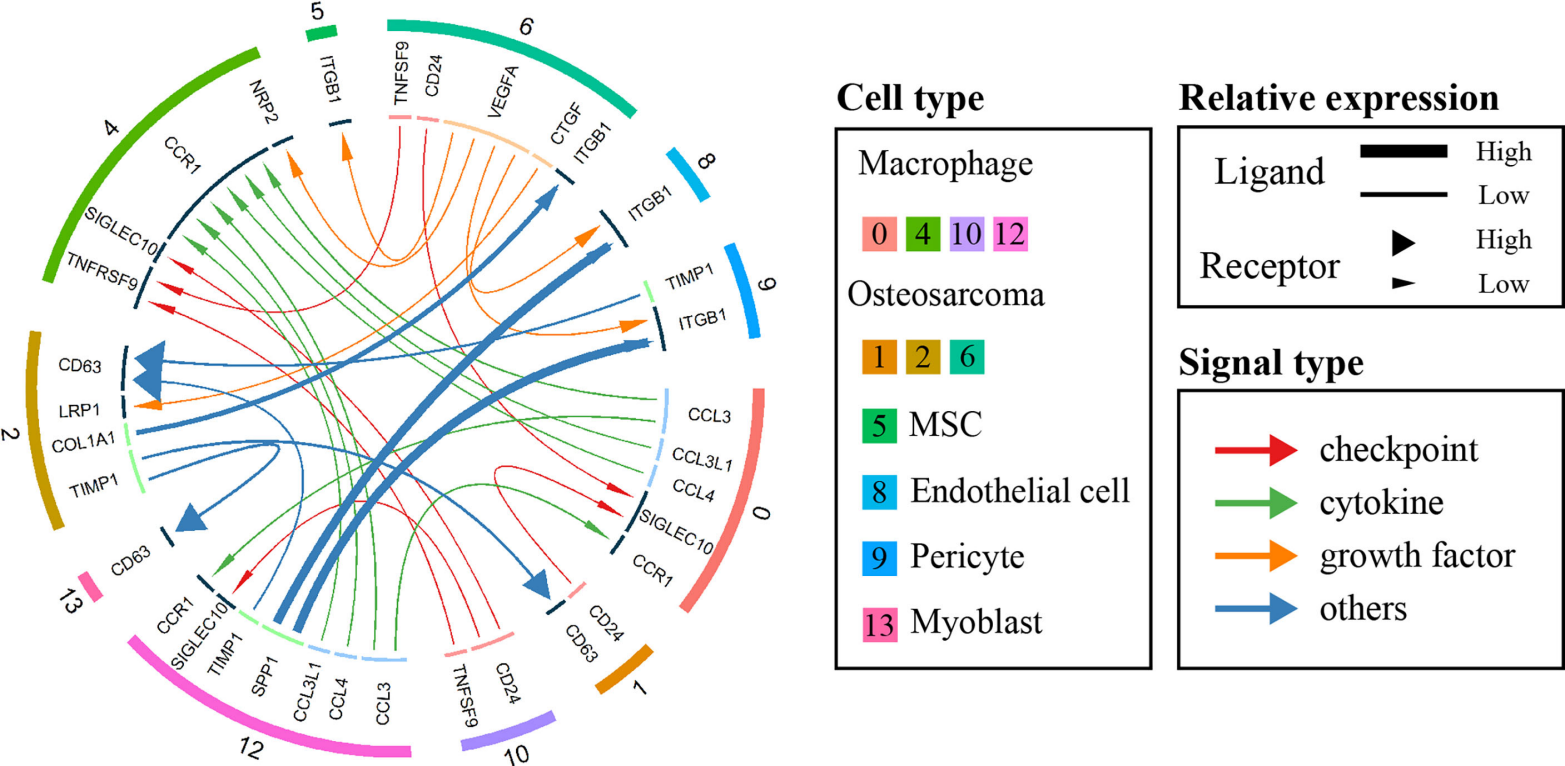
Circos plot showing cellular crosstalk within the OS microenvironment. The outside ring of the circos plot displays cell clusters, and the inside shows the details of each interacting ligand-receptor pair. The lines and arrowheads inside the circos plot were scaled to indicate the relative signal strength of the ligand and receptor, respectively, and different colors and types of lines were used to illustrate various categories.

To investigate and visualize the cell-cell interaction network in the OS microenvironment, the iTALK package of R was used to analyze the ligand-receptor interactions in different cell types (23). Based on the primary function of the ligand, iTALK further classifies intercellular crosstalk into 4 categories: checkpoint, cytokine, growth factor and other. In terms of the checkpoint category, OS cells of clusters 1 and 6 expressed higher levels of CD24, which could interact with the SIGLEC10 receptor of cluster 0 macrophages to regulate the antitumor immune response of macrophages and promote immune evasion (26). The cytokine category showed internal connections among macrophages. CCL3, CCL4 and CCL3L1 secreted by cluster 0 and 12 macrophages could interact with CCR1, which was highly expressed on the surface of cluster 4 macrophages, promoted their migration, and regulated the inflammatory response (27). Regarding the growth factor category, we also found that cluster 6 OS cells likely produce VEGFA, which binds to ITGB1 receptors on endothelial cells (ECs) to regulate their migration and promote new vessel formation and tumor metastasis (28–30). (**Figure 2**)

### Identification of Different Invasive Osteosarcoma Cells

**Figure 3:**
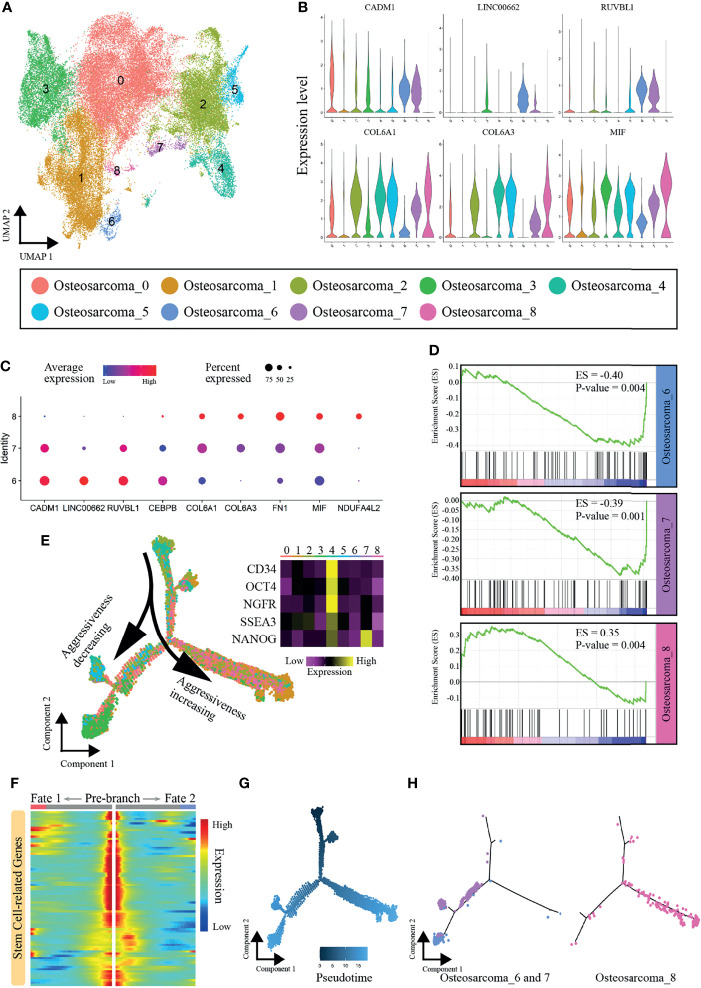
Heterogeneity of OS cells. **(A)** Nine main OS cell subclusters were identified by UMAP analysis. **(B)** Violin plot showing expression levels of some vital genes that promoted or inhibited OS metastasis. **(C)** Dot plots showing expression of the nine signature genes across the nine subcellular clusters. **(D)** GSEA results regarding enrichment of the PI3K-AKT signaling pathway in subclusters 6, 7 and 8. **(E)** Pseudotemporal trajectory plot showing the dynamics of OS subclusters. **(F)** Heatmap showing expression alterations of stem cell-related genes under different cell fates defined by pseudotime analysis. **(G)** Pseudotime of OS cells. **(H)** Differentiation trajectories of subclusters 6, 7 and 8.

To study the heterogeneity among OS cells and find new cell subpopulations closely related to OS metastasis, data for 53,441 OS cells were extracted, and the cells were clustered into nine subclusters (named Osteosarcoma_0 ~ Osteosarcoma_8), and Osteosarcoma_3 and 8 were chondroblastic and the rest were osteoblastic cells (**Figure 3A** and **Figure S1**). As illustrated in **Figures 3B, C**, the genes CADM1, LINC00662, and RUVBL1, which have been shown to inhibit OS metastasis (31–33), were highly expressed by the cells in subclusters 6 and 7. In addition, Osteosarcoma_8 cells showed high expression levels of COL6A1, COL6A3 and MIF, and these genes have been confirmed to be associated with metastasis (34–37). In addition, many genes that play important roles in osteosarcoma display distinct expression patterns between Osteosarcoma_6, 7 and 8 (**Figure S2A**).

PI3K-AKT signaling is one of the key pathways promoting the metastasis in OS, and inhibition of PI3K-AKT signaling yields enhanced antitumor activity, impaired tumor growth, and reduced migratory and metastatic potential (38, 39). The GSEA results revealed that the PI3K-AKT pathway was activated in Osteosarcoma_8 (ES=0.35, P-value=0.004) but was inhibited in Osteosarcoma_6 (ES=-0.40, P-value=0.004) and Osteosarcoma_7 (ES=-0.39, P-value=0.001) (**Figure 3D**). We further constructed an osteosarcoma-related gene set using the results of the edgeR package and performed GSEA enrichment analysis. Similarly, the results also confirmed that there were significant differences in cell states between Osteosarcoma _6, 7 and 8 (**Figure S2B**). Thus, we demonstrated that subcluster 8 contained aggressive cells, while the cells in subclusters 6 and 7 showed limited associations with metastasis.

### Defining the Transcriptional Dynamics of Osteosarcoma

Next, we explored the dynamics and regulators of cell fate decisions in OS cells with Monocle 2. The pseudotime trajectory was visualized and is shown in **Figure 3E**. Strikingly, we noticed that Osteosarcoma_4 was mainly located at the beginning of the trajectory path, with high expression of many stem and progenitor cell marker genes (CD34, Oct-4, NGFR, SSEA3 and Nanog) (40–44). A set of stem cell-related genes were collected from the “CellMarker” database (http://biocc.hrbmu.edu.cn/CellMarker/index.jsp), and their expression gradually decreased with the cell trajectory (**Figure 3F**). Therefore, Osteosarcoma_4 was identified as the principal progenitor. As cells progressed along a differentiation trajectory, they diverged along two separate paths, which represented two distinctive cell fates (**Figures 3E–G**). Interestingly, Osteosarcoma_8, which represented the more aggressive subpopulation, mainly followed the right differentiation trajectory after the branch point. In contrast, the metastatically indolent OS cells in Osteosarcoma_6 and Osteosarcoma_7 predominantly followed the left trajectory (**Figure 3H**). In summary, Osteosarcoma_8 OS cells showed different differentiation states from the cells of Osteosarcoma_6 and Osteosarcoma_7, which may be the reason for their different levels of invasiveness.

### Establishment of the Risk Regression Model

**Figure 4:**
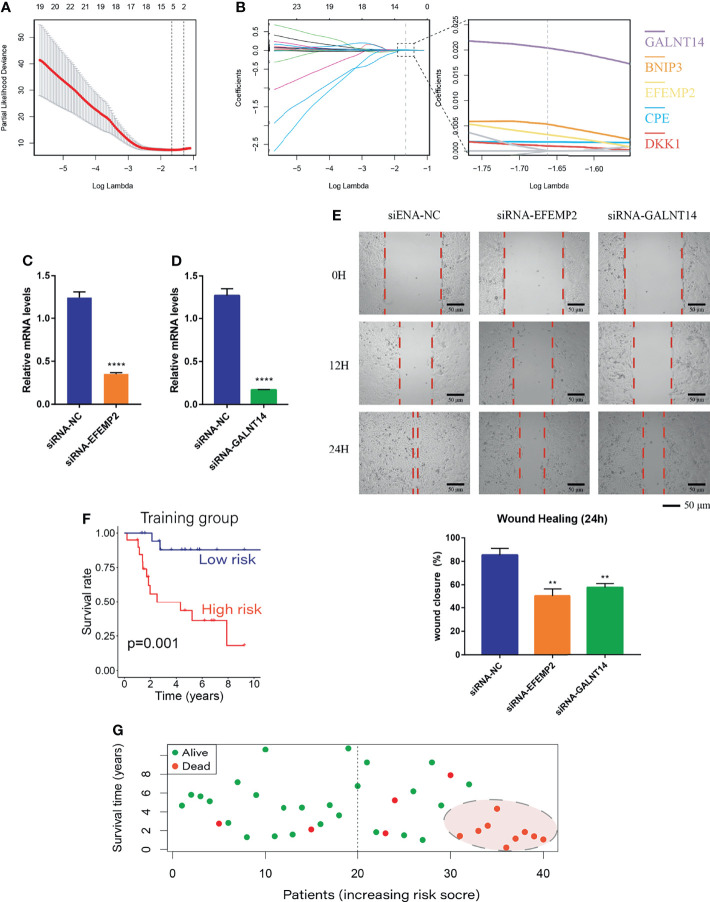
Establishment of the BCDEG model. **(A)** The error rate for model cross-validation. Each lambda value with error bars gives a confidence interval for the cross-validation error rate. The vertical dashed line on the left represents the minimum error, and the number of genes in each model is given at the top of the plot. **(B)** A model considering prognosis-related genes was produced by maximizing the appropriate penalized log-likelihood. Lasso penalty leads to convergence of gene coefficients, and the colored line shows the trend of gene coefficients as a function of lambda. The number of genes with nonzero regression coefficients is shown at the top of the plot. **(C)** siRNA-EFEMP2 significantly inhibited the expression of EFEMP2. **(D)** siRNA-GALNT14 significantly inhibited the expression of GALNT14. **(E)** Degree of wound healing at 0, 12 and 24 hours; the red dotted lines represent the edge of the scratch. **(F)** Survival curve for the training group. The prognosis of the low-risk group was better than that of the high-risk group. **(G)** Survival status plot of the training group ranked according to risk score. **p < 0.01 and ****p < 0.0001.

Differential analysis was performed between weakly metastatic subclusters (Osteosarcoma_6 and Osteosarcoma_7) and the highly invasive subcluster (Osteosarcoma_8) of OS cells, and 813 metastasis-related genes were identified. As described in Materials and Methods, BCDEG, a model considering five metastasis-related genes (GALNT14, BNIP3, EFEMP2, CPE and DKK1), was developed (**Figures 4A, B**). Previous studies have confirmed that silencing BNIP3 and DKK1 can effectively inhibit OS proliferation and promote apoptosis (45–47) and that CPE overexpression enhances OS growth, migration, and invasion (48, 49). Since the roles of EFEMP2 and GALNT14 in OS have not been explored, which is worthy of further study. We used siRNA to significantly suppress the expression of EFEMP2 and GALNT14 (**Figures 4C, D**). Wound healing analysis showed that, within 24 hours, the scratches in the negative control group were close to healing, while those in the siRNA-EFEMP2 and the siRNA-GALNT14 groups were still relatively obvious (**Figures 4E**). These findings suggest that inhibiting EFEMP2 and GALNT14 could decrease the invasion and metastasis ability of OS.

The BCDEG model was applied to predict survival in the training group, and the results of Kaplan-Meier analysis showed that the risk score was significantly and negatively associated with the prognosis of OS patients (**Figure 4F**). When patients were divided into groups according to risk score, the mortality rate was significantly higher in the high-risk group than the low-risk group, and almost all patients with the highest risk scores died with a very short survival period (**Figure 4G**, P=0.001).

### Excellent Performance of BCDEG

**Figure 5:**
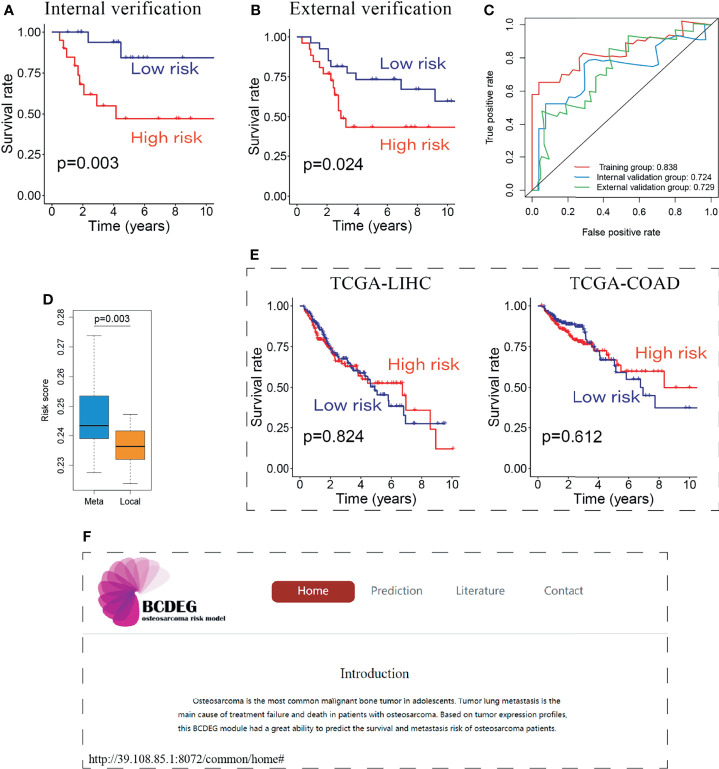
Evaluation of the BCDEG model. **(A)** Survival curve for the internal verification group. **(B)** Survival curve for the external verification group. **(C)** Time-dependent ROC curves for three groups. The AUCs of the 3 groups were all greater than 0.7. **(D)** Box plot showing the difference in risk score between metastatic and localized OS patients. **(E)** Survival curve for the BCDEG model to predict the prognosis of liver hepatocellular carcinoma and colon adenocarcinoma patients. **(F)** Snapshots of the BCDEG web app.

The remaining patients were used to verify the BCDEG model, and similar results were observed in the internal (**Figure 5A**) and external validation groups (**Figure 5B**). Time-dependent ROC curves were produced to assess the prognostic ability of BCDEG, and the AUCs at 5 years were 0.838, 0.724, and 0.729 in the training, internal validation, and external validation groups, respectively (**Figure 5C**), indicating that the BCDEG model had a great ability to predict the survival of OS patients. We also wondered whether BCDEG could predict OS metastasis. We generated a box plot, which showed that the risk scores of metastatic patients were significantly higher than those of localized OS patients (**Figure 5D**), and the sensitivity and specificity of prediction were 0.81 and 0.74, respectively.

We also applied the BCDEG model for patients with liver hepatocellular carcinoma and colon adenocarcinoma from The Cancer Genome Atlas (TCGA). The results of TCGA-LIHC and TCGA-COAD stand in sharp contrast to OS patients, as that there were no significant differences between the survival of high-risk and low-risk groups (**Figure 5E**), indicating that this model is specific for OS.

To enable clinicians to have convenient access to this model, an open-source web app was generated. Users can calculate multiple patient risk scores online to evaluate prognosis and guide clinical treatment. The web app can be accessed at http://39.108.85.1:8072/common/home (**Figure 5F**).

## Discussion

OS is the most common primary malignant bone tumor in adolescents and children. A high rate of distant metastasis is its characteristic feature, and the occurrence of metastasis has a great impact on the prognosis of patients (50). The rapid development of scRNA-seq technology has enabled research on highly heterogeneous tumors, including OS, which is helpful for understanding the tumor microenvironment and exploring new cell populations (51).

In this study, we identified alterations in single OS cells through comprehensive analysis of two different types of sequencing data, isolated 14 major cell populations in OS tissue, and annotated 8 cell types by extracting characteristic genes. By applying intercellular communication analysis software, we collected 26 reliable ligand-receptor signaling cell pairs and characterized the complex regulatory network in the multicellular tumor microenvironment. OS cells act on SIGLEC10 on macrophages and the ITGB1 receptor on the surface of ECs to achieve immune escape and promote angiogenesis (26, 52, 53), which provides a new therapeutic approach for inhibiting the growth and metastasis of OS.

Deeply exploring the high heterogeneity of OS cells and finding new subpopulations with different biological functions have become goals of research (54–56). We further subdivided OS cells into 9 subpopulations. One subpopulation was highly invasive, whereas two were found to be metastatically inert by annotation of metastasis-related markers and pathway enrichment analysis. The developmental trajectory analysis showed that these two kinds of subpopulations had different differentiation fates during development. A highly aggressive subpopulation is often considered to play a key role in OS metastasis (57). Exploring the mechanisms underlying the origination and development of this population, further studying how to regulate or change the differentiation fate of OS cells, and promoting the differentiation of tumor cells toward a phenotype with low invasiveness are of great significance for effectively inhibiting the metastasis of OS and improving the prognosis of patients.

ScRNA-seq gets more detailed information at the cellular level, and bulk RNA-seq and scRNA-seq have certain consistency. Combining the scRNA-seq with existing bulk data from large cohorts can help decipher the molecular mechanisms of OS progression and metastasis (58). Based on the above research results, we established a metastasis-associated risk assessment model for OS, which considered five genes: BNIP3, CPE, DKK1, EFEMP2 and GALNT14. The excellent effectiveness and specificity of this model were demonstrated not only in the training group, internal and external validation groups, but also *via* validation in patients with other types of tumors. Sheen et al. (13) developed an OS metastatic risk prediction model using metabolic imaging phenotypes, but its sensitivity was only 0.63. In general, sensitivity is important for OS risk models because metastasis is extremely unfavorable for the prognosis of OS patients, and the BCDEG model improves this situation. Overall, the model could effectively predict prognosis and evaluate the risk of tumor metastasis, providing a theoretical basis for the development of personalized diagnosis and treatment plans for patients.

In terms of the limitations of these data, there were relatively few OS scRNA-seq samples, and there was a lack of corresponding normal tissue samples. Analysis of additional tumor and normal samples would be conducive to eliminating the heterogeneity caused by individual differences. Nevertheless, we provide moderate evidence that there are strong and meaningful associations between different cell types in the OS microenvironment, with different subpopulations of OS cells performing different biological functions.

In summary, our research in-depth analyzed RNA-seq and bulk RNA-seq data in a complementary way to explore the intercellular relationships and internal mechanisms that affect the occurrence, progression and metastasis of OS. Moreover, BCDEG, an effective, reliable and easy-to-use prognostic model, was established. These results will aid in accurate treatment of OS and further improve the therapeutic effect and 5-year survival rate of patients.

## Data Availability Statement

The original contributions presented in the study are included in the article/**Supplementary Material**. Further inquiries can be directed to the corresponding authors.

## Author Contributions

Conceptualization, KT and TL. Methodology, KT and CY. Software, JG (1st author). Validation, JG (1st author) and HT. Formal analysis, HT and PH. Investigation, JG (4th author). Resources, YS. Data curation, JG (4th author) and PH. Writing-original draft preparation, JG (1st author) and JG (4th author). Writing-review and editing, KT. Visualization, JG (1st author) and TL. Supervision, CY. Project administration, TL. Funding acquisition, KT. All authors contributed to the article and approved the submitted version.

## Funding

This study was supported by the Personalization Training Program for the Training Object of the Outstanding Talents of Army Medical University (2020, 4139Z2C2) and Chief Medical Expert of Chongqing (2019, 414Z333).

## Conflict of Interest

The authors declare that the research was conducted in the absence of any commercial or financial relationships that could be construed as a potential conflict of interest.

## Publisher’s Note

All claims expressed in this article are solely those of the authors and do not necessarily represent those of their affiliated organizations, or those of the publisher, the editors and the reviewers. Any product that may be evaluated in this article, or claim that may be made by its manufacturer, is not guaranteed or endorsed by the publisher.

## Abbreviations

scRNA-seq, single-cell RNA sequencing; SNN, shared nearest neighbor; UMAP, uniform manifold approximation and projection; GSEA, gene set enrichment analysis; ES, enrichment score; DDRTree, discriminative dimensionality reduction with trees; TCGA, The Cancer Genome Atlas.

## References

[1] Kansara M Teng MW Smyth MJ Thomas DM. Translational Biology of Osteosarcoma. Nat Rev Cancer (2014) 14:722–35. doi: 10.1038/nrc3838 25319867

[2] Bousquet M Noirot C Accadbled F Sales De Gauzy J Castex MP Brousset P. Whole-Exome Sequencing in Osteosarcoma Reveals Important Heterogeneity of Genetic Alterations. Ann Oncol (2016) 27:738–44. doi: 10.1093/annonc/mdw009 26787232

[3] Ho XD Nguyen HG Trinh LH Reimann E Prans E Kõks G. Analysis of the Expression of Repetitive DNA Elements in Osteosarcoma. Front Genet (2017) 8:193. doi: 10.3389/fgene.2017.00193 29250102 PMC5714928

[4] Song Y An W Wang H Gao Y Han J Hao C. LRH1 Acts as an Oncogenic Driver in Human Osteosarcoma and Pan-Cancer. Front Cell Dev Biol (2021) 9:643522. doi: 10.3389/fcell.2021.643522 33791301 PMC8005613

[5] Ekhtiari S Chiba K Popovic S Crowther R Wohl G Kin on Wong A. First Case of Osteosarcoma in a Dinosaur: A Multimodal Diagnosis. Lancet Oncol (2020) 21:1021–2. doi: 10.1016/S1470-2045(20)30171-6 32758461

[6] Ritter J Bielack SS. Osteosarcoma. Ann Oncol (2010) 21(Suppl 7):vii320–5. doi: 10.1093/annonc/mdq276 20943636

[7] Rothzerg E Ho XD Xu J Wood D Märtson A Kõks S. Upregulation of 15 Antisense Long Non-Coding RNAs in Osteosarcoma. Genes (Basel) (2021) 12:1132. doi: 10.3390/genes12081132 34440306 PMC8394133

[8] Bernardini G Laschi M Geminiani M Santucci A. Proteomics of Osteosarcoma. Expert Rev Proteomics (2014) 11:331–43. doi: 10.1586/14789450.2014.900445 24654989

[9] Yao Z Han L Chen Y He F Sun B Kamar S. Hedgehog Signalling in the Tumourigenesis and Metastasis of Osteosarcoma, and Its Potential Value in the Clinical Therapy of Osteosarcoma. Cell Death Dis (2018) 9:701. doi: 10.1038/s41419-018-0647-1 29899399 PMC5999604

[10] Zhao J Dean DC Hornicek FJ Yu X Duan Z. Emerging Next-Generation Sequencing-Based Discoveries for Targeted Osteosarcoma Therapy. Cancer Lett (2020) 474:158–67. doi: 10.1016/j.canlet.2020.01.020 31987920

[11] Chen R Wang G Zheng Y Hua Y Cai Z. Long non-Coding RNAs in Osteosarcoma. Oncotarget (2017) 8:20462–75. doi: 10.18632/oncotarget.14726 PMC5386777 28103585

[12] Wang D Niu X Wang Z Song CL Huang Z Chen KN. Multiregion Sequencing Reveals the Genetic Heterogeneity and Evolutionary History of Osteosarcoma and Matched Pulmonary Metastases. Cancer Res (2019) 79:7–20. doi: 10.1158/0008-5472.CAN-18-1086 30389703

[13] Sheen H Kim W Byun BH Kong CB Song WS Cho WH. Metastasis Risk Prediction Model in Osteosarcoma Using Metabolic Imaging Phenotypes: A Multivariable Radiomics Model. PloS One (2019) 14:e0225242. doi: 10.1371/journal.pone.0225242 31765423 PMC6876771

[14] Flores RJ Kelly AJ Li Y Nakka M Barkauskas DA Krailo M. A Novel Prognostic Model for Osteosarcoma Using Circulating CXCL10 and FLT3LG. Cancer (2017) 123:144–54. doi: 10.1002/cncr.30272 PMC5161556 27529817

[15] Jafari F Javdansirat S Sanaie S Naseri A Shamekh A Rostamzadeh D. Osteosarcoma: A Comprehensive Review of Management and Treatment Strategies. Ann Diagn Pathol (2020) 49:151654. doi: 10.1016/j.anndiagpath.2020.151654 33130384

[16] Dean DC Shen S Hornicek FJ Duan Z. From Genomics to Metabolomics: Emerging Metastatic Biomarkers in Osteosarcoma. Cancer Metastasis Rev (2018) 37:719–31. doi: 10.1007/s10555-018-9763-8 30167827

[17] Fan J Slowikowski K Zhang F. Single-Cell Transcriptomics in Cancer: Computational Challenges and Opportunities. Exp Mol Med (2020) 52:1452–65. doi: 10.1038/s12276-020-0422-0 PMC8080633 32929226

[18] Liu J Xu T Jin Y Huang B Zhang Y. Progress and Clinical Application of Single-Cell Transcriptional Sequencing Technology in Cancer Research. Front Oncol (2020) 10:593085. doi: 10.3389/fonc.2020.593085 33614479 PMC7886993

[19] Brown HK Tellez-Gabriel M Heymann D. Cancer Stem Cells in Osteosarcoma. Cancer Lett (2017) 386:189–95. doi: 10.1016/j.canlet.2016.11.019 27894960

[20] Li H. Single-Cell RNA Sequencing in Drosophila: Technologies and Applications. Wiley Interdiscip Rev Dev Biol (2020) 10:e396. doi: 10.1002/wdev.396 32940008 PMC7960577

[21] Hao Y Hao S Andersen-Nissen E Mauck WM Zheng S Butler A. Integrated Analysis of Multimodal Single-Cell Data. Cell (2021) 184:3573–87.e29. doi: 10.1016/j.cell.2021.04.048 PMC8238499 34062119

[22] Korsunsky I Millard N Fan J Slowikowski K Zhang F Wei K. Fast, Sensitive and Accurate Integration of Single-Cell Data With Harmony. Nat Methods (2019) 16:1289–96. doi: 10.1038/s41592-019-0619-0 PMC6884693 31740819

[23] Wang Y Wang R Zhang S Song S Jiang C Han G. iTALK: An R Package to Characterize and Illustrate Intercellular Communication. bioRxiv (2019) 507871. doi: 10.1101/507871

[24] Qiu X Hill A Packer J Lin D Ma YA Trapnell C. Single-Cell mRNA Quantification and Differential Analysis With Census. Nat Methods (2017) 14:309–15. doi: 10.1038/nmeth.4150 PMC5330805 28114287

[25] Schneider CA Rasband WS Eliceiri KW. NIH Image to ImageJ: 25 Years of Image Analysis. Nat Methods (2012) 9:671–5. doi: 10.1038/nmeth.2089 PMC5554542 22930834

[26] Barkal AA Brewer RE Markovic M Kowarsky M Barkal SA Zaro BW. CD24 Signalling Through Macrophage Siglec-10 Is a Target for Cancer Immunotherapy. Nature (2019) 572:392–6. doi: 10.1038/s41586-019-1456-0 PMC6697206 31367043

[27] Wang J Tian Y Phillips KL Chiverton N Haddock G Bunning RA. Tumor Necrosis Factor α- and Interleukin-1β-Dependent Induction of CCL3 Expression by Nucleus Pulposus Cells Promotes Macrophage Migration Through CCR1. Arthritis Rheum (2013) 65:832–42. doi: 10.1002/art.37819 PMC3582738 23233369

[28] Cheng X Liu Y Chu H Kao HY. Promyelocytic Leukemia Protein (PML) Regulates Endothelial Cell Network Formation and Migration in Response to Tumor Necrosis Factor α (Tnfα) and Interferon α (Ifnα). J Biol Chem (2012) 287:23356–67. doi: 10.1074/jbc.M112.340505 PMC3390613 22589541

[29] Chen MB Lamar JM Li R Hynes RO Kamm RD. Elucidation of the Roles of Tumor Integrin β1 in the Extravasation Stage of the Metastasis Cascade. Cancer Res (2016) 76:2513–24. doi: 10.1158/0008-5472.CAN-15-1325 PMC4873393 26988988

[30] Lu Q Xie Z Yan C Ding Y Ma Z Wu S. SNRK (Sucrose Nonfermenting 1-Related Kinase) Promotes Angiogenesis *In Vivo* . Arterioscler Thromb Vasc Biol (2018) 38:373–85. doi: 10.1161/ATVBAHA.117.309834 PMC5785416 29242271

[31] Cai H Miao M Wang Z. miR-214-3p Promotes the Proliferation, Migration and Invasion of Osteosarcoma Cells by Targeting CADM1. Oncol Lett (2018) 16:2620–8. doi: 10.3892/ol.2018.8927 PMC6036594 30013657

[32] Chen J Liu G Wu Y Ma J Wu H Xie Z. CircMYO10 Promotes Osteosarcoma Progression by Regulating miR-370-3p/RUVBL1 Axis to Enhance the Transcriptional Activity of β-Catenin/LEF1 Complex *via* Effects on Chromatin Remodeling. Mol Cancer (2019) 18:150. doi: 10.1186/s12943-019-1076-1 31665067 PMC6819556

[33] Liu S Meng X. LINC00662 Long Non-Coding RNA Knockdown Attenuates the Proliferation, Migration, and Invasion of Osteosarcoma Cells by Regulating the microRNA-15a-5p/Notch2 Axis. Onco Targets Ther (2020) 13:7517–30. doi: 10.2147/OTT.S256464 PMC7429411 32848412

[34] Ho XD Phung P Le VQ Nguyen V,H Reimann E Prans E. Whole Transcriptome Analysis Identifies Differentially Regulated Networks Between Osteosarcoma and Normal Bone Samples. Exp Biol Med (Maywood) (2017) 242:1802–11. doi: 10.1177/1535370217736512 PMC5714151 29050494

[35] Wang C Zhou X Li W Li M Tu T Ba X. Macrophage Migration Inhibitory Factor Promotes Osteosarcoma Growth and Lung Metastasis Through Activating the RAS/MAPK Pathway. Cancer Lett (2017) 403:271–9. doi: 10.1016/j.canlet.2017.06.011 28642171

[36] Guo HL Chen G Song ZL Sun J Gao XH Han YX. COL6A3 Promotes Cellular Malignancy of Osteosarcoma by Activating the PI3K/AKT Pathway. Rev Assoc Med Bras (1992) (2020) 66:740–5. doi: 10.1590/1806-9282.66.6.740 32696868

[37] Zhang Y Liu Z Yang X Lu W Chen Y Lin Y. H3K27 Acetylation Activated-COL6A1 Promotes Osteosarcoma Lung Metastasis by Repressing STAT1 and Activating Pulmonary Cancer-Associated Fibroblasts. Theranostics (2021) 11:1473–92. doi: 10.7150/thno.51245 PMC7738898 33391546

[38] Ramaswamy B Lu Y Teng KY Nuovo G Li X Shapiro CL. Hedgehog Signaling is a Novel Therapeutic Target in Tamoxifen-Resistant Breast Cancer Aberrantly Activated by PI3K/AKT Pathway. Cancer Res (2012) 72:5048–59. doi: 10.1158/0008-5472.CAN-12-1248 PMC3837449 22875023

[39] Behjati S Tarpey PS Haase K Ye H Young MD Alexandrov LB. Recurrent Mutation of IGF Signalling Genes and Distinct Patterns of Genomic Rearrangement in Osteosarcoma. Nat Commun (2017) 8:15936. doi: 10.1038/ncomms15936 28643781 PMC5490007

[40] Pan GJ Chang ZY Schöler HR Pei D. Stem Cell Pluripotency and Transcription Factor Oct4. Cell Res (2002) 12:321–9. doi: 10.1038/sj.cr.7290134 12528890

[41] Chambers I Colby D Robertson M Nichols J Lee S Tweedie S. Functional Expression Cloning of Nanog, a Pluripotency Sustaining Factor in Embryonic Stem Cells. Cell (2003) 113:643–55. doi: 10.1016/S0092-8674(03)00392-1 12787505

[42] Zhuo J Fu W Liu S. Correlation of Contrast-Enhanced Ultrasound With Two Distinct Types of Blood Vessels for the Assessment of Angiogenesis in Lewis Lung Carcinoma. Ultraschall Med (2014) 35:468–72. doi: 10.1055/s-0033-1356194 24327471

[43] Cheung SK Chuang PK Huang HW Hwang-Verslues WW Cho CH Yang WB. Stage-Specific Embryonic Antigen-3 (SSEA-3) and β3galt5 are Cancer Specific and Significant Markers for Breast Cancer Stem Cells. Proc Natl Acad Sci USA (2016) 113:960–5. doi: 10.1073/pnas.1522602113 PMC4743801 26677875

[44] Hicks MR Hiserodt J Paras K Fujiwara W Eskin A Jan M. ERBB3 and NGFR Mark a Distinct Skeletal Muscle Progenitor Cell in Human Development and hPSCs. Nat Cell Biol (2018) 20:46–57. doi: 10.1038/s41556-017-0010-2 29255171 PMC5962356

[45] Zhao X Sun S Xu J Luo Y Xin Y Wang Y. MicroRNA-152 Inhibits Cell Proliferation of Osteosarcoma by Directly Targeting Wnt/β-Catenin Signaling Pathway in a DKK1-Dependent Manner. Oncol Rep (2018) 40:767–74. doi: 10.3892/or.2018.6456 29845282

[46] Li S Liu F Pei Y Wang W Zheng K Zhang X. Long Noncoding RNA TTN-AS1 Enhances the Malignant Characteristics of Osteosarcoma by Acting as a Competing Endogenous RNA on microRNA-376a Thereby Upregulating Dickkopf-1. Aging (Albany NY) (2019) 11:7678–93. doi: 10.18632/aging.102280 PMC6781980 31525734

[47] He G Pan X Liu X Zhu Y Ma Y Du C. HIF-1α-Mediated Mitophagy Determines ZnO Nanoparticle-Induced Human Osteosarcoma Cell Death Both *In Vitro* and *In Vivo* . ACS Appl Mater Interfaces (2020) 12:48296–309. doi: 10.1021/acsami.0c12139 33054172

[48] Fan S Li X Li L Wang L Du Z Yang Y. Silencing of Carboxypeptidase E Inhibits Cell Proliferation, Tumorigenicity, and Metastasis of Osteosarcoma Cells. Onco Targets Ther (2016) 9:2795–803. doi: 10.2147/OTT.S98991 PMC4869623 27274275

[49] Fan S Gao X Chen P Li X. Carboxypeptidase E-ΔN Promotes Migration, Invasiveness, and Epithelial-Mesenchymal Transition of Human Osteosarcoma Cells *via* the Wnt-β-Catenin Pathway. Biochem Cell Biol (2019) 97:446–53. doi: 10.1139/bcb-2018-0236 30508384

[50] Wu X Yan L Liu Y Shang L. Circ_0000527 Promotes Osteosarcoma Cell Progression Through Modulating miR-646/ARL2 Axis. Aging (Albany NY) (2021) 13:6091–102. doi: 10.18632/aging.202602 PMC7950279 33617480

[51] Lim B Lin Y Navin N. Advancing Cancer Research and Medicine With Single-Cell Genomics. Cancer Cell (2020) 37:456–70. doi: 10.1016/j.ccell.2020.03.008 PMC7899145 32289270

[52] Park H Choi HJ Kim J Kim M Rho SS Hwang D. Homeobox D1 Regulates Angiogenic Functions of Endothelial Cells *via* Integrin β1 Expression. Biochem Biophys Res Commun (2011) 408:186–92. doi: 10.1016/j.bbrc.2011.04.017 21501586

[53] Cui Y Fu S Sun D Xing J Hou T Wu X. EPC-Derived Exosomes Promote Osteoclastogenesis Through LncRNA-Malat1. J Cell Mol Med (2019) 23:3843–54. doi: 10.1111/jcmm.14228 PMC6533478 31025509

[54] Scott MC Tomiyasu H Garbe JR Cornax I Amaya C O’sullivan MG. Heterotypic Mouse Models of Canine Osteosarcoma Recapitulate Tumor Heterogeneity and Biological Behavior. Dis Model Mech (2016) 9:1435–44. doi: 10.1242/dmm.026849 PMC5200896 27874835

[55] Hatina J Kripnerova M Houfkova K Pesta M Kuncova J Sana J. Sarcoma Stem Cell Heterogeneity. Adv Exp Med Biol (2019) 1123:95–118. doi: 10.1007/978-3-030-11096-3_7 31016597

[56] Schiavone K Garnier D Heymann MF Heymann D. The Heterogeneity of Osteosarcoma: The Role Played by Cancer Stem Cells. Adv Exp Med Biol (2019) 1139:187–200. doi: 10.1007/978-3-030-14366-4_11 31134502

[57] Martins-Neves SR Lopes Á O Do Carmo A Paiva AA Simões PC Abrunhosa AJ. Therapeutic Implications of an Enriched Cancer Stem-Like Cell Population in a Human Osteosarcoma Cell Line. BMC Cancer (2012) 12:139. doi: 10.1186/1471-2407-12-139 22475227 PMC3351999

[58] Zhang L Zhang Y Wang C Yang Y Ni Y Wang Z. Integrated Single-Cell RNA Sequencing Analysis Reveals Distinct Cellular and Transcriptional Modules Associated With Survival in Lung Cancer. Signal Transduct Target Ther (2022) 7:9. doi: 10.1038/s41392-021-00824-9 35027529 PMC8758688

