# Supplementary material for: Single-Cell Profiling of Tumor Microenvironment Heterogeneity in Osteosarcoma Identifies a Highly Invasive Subcluster for Predicting Prognosis
Source: Front Oncol. 2022 Apr 6;12:732862. doi: 10.3389/fonc.2022.732862 (PMC9020875; doi:10.3389/fonc.2022.732862)
Supplement: Supplementary file 1 [file DataSheet_1.docx]

Single-cell profiling of tumor microenvironment heterogeneity in osteosarcoma identifies a highly invasive subcluster for predicting prognosis

**Supplementary Information**

**Supplementary Figure 1**


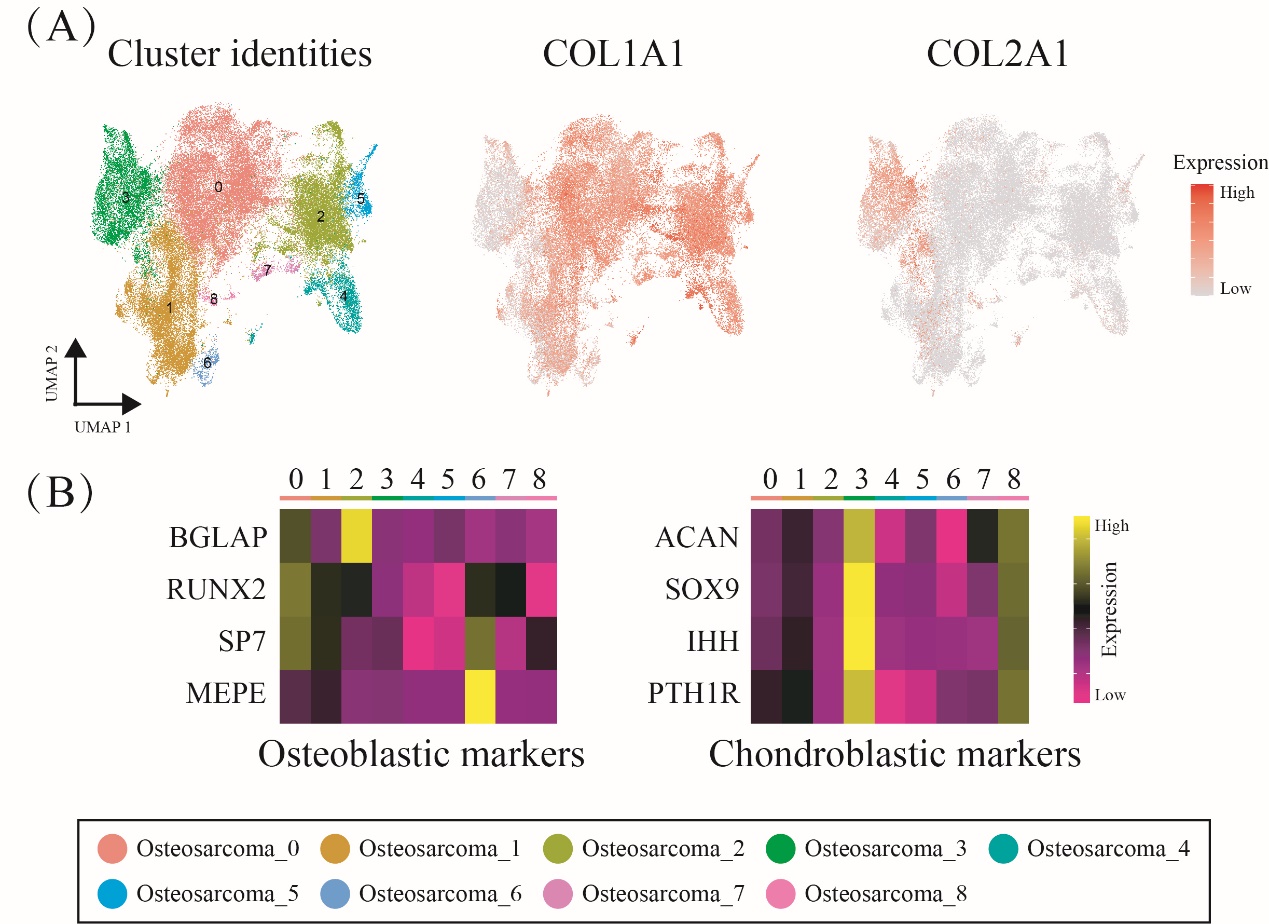


**Supplementary Fig. 1.** Expression level of osteoblastic and chondroblastic genes of the distinct osteosarcoma subclusters. A. Identification of osteosarcoma cell subclusters and the expression patterns of COL1A1 and COL2A1 in distinct subclusters. B. Heatmap showing expression levels of some identify marker genes in different osteosarcoma subsets.

**Supplementary Figure 2**


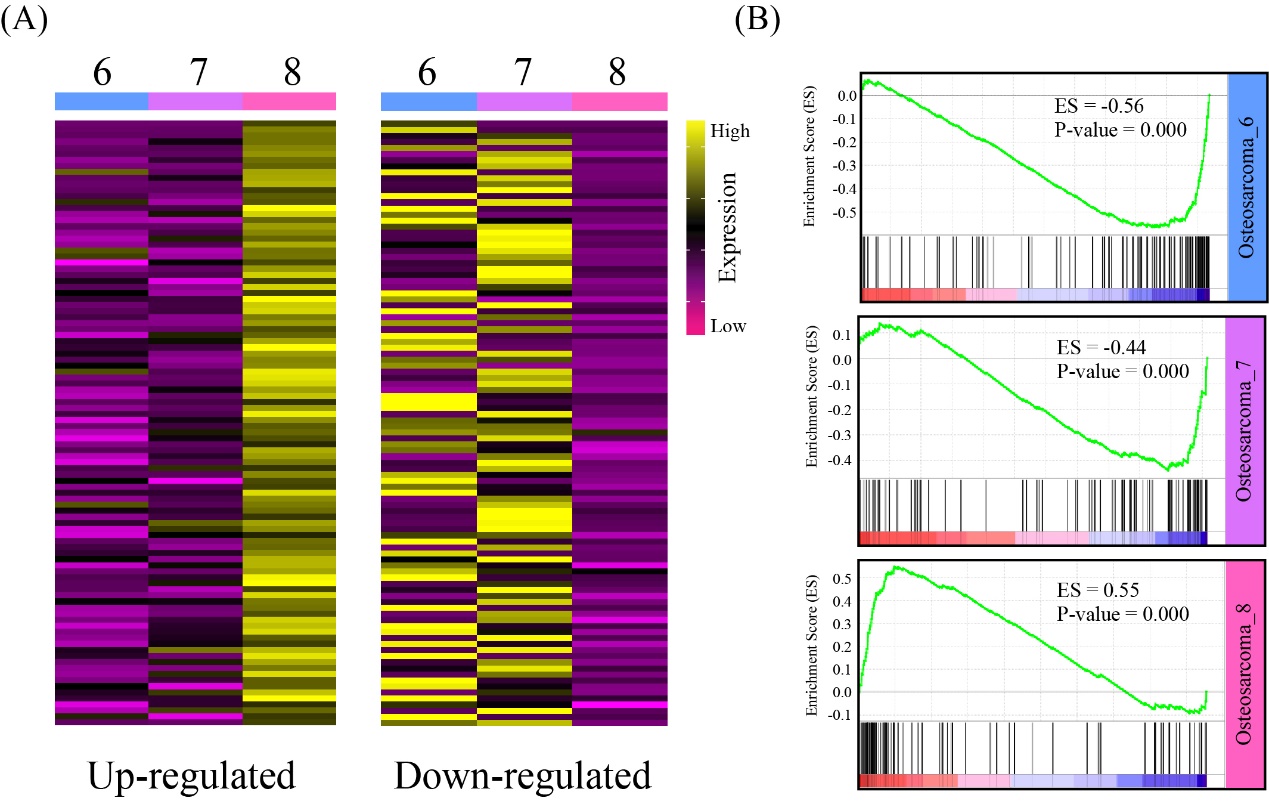


**Supplementary Fig. 2.** Osteosarcoma_8 exhibits unique cellular properties. A. Expression patterns of core genes in osteosarcoma. Left: up-regulated genes in osteosarcoma are highly expressed in Osteosarcoma_8. Right: down- regulated genes in osteosarcoma are low expressed in Osteosarcoma_8. B. GSEA results regarding enrichment of the osteosarcoma-related gene set in subclusters 6, 7 and 8.

**Supplementary Table 1.** The canonical markers for the 14 cell clusters in osteosarcoma tissues.

| cluster identity | cell type | marker genes | References |
| --- | --- | --- | --- |
| 0 | Macrophages | HLA-DRA, CD74, AIF1 | [1-3] |
| 1 | Osteosarcoma cells | COL1A1, RUNX2, SP7 | [4] |
| 2 | Osteosarcoma cells | CXCL12, MMP13, POSTN | [4, 5] |
| 3 | Osteosarcoma cells | MEPE, COL11A1, MYC | [6-8] |
| 4 | Macrophages | CD68, CSF1R, SPI1 | [9, 10] |
| 5 | Mesenchymal stem cells | NES, HMGB2, CCNB2 | [4, 11, 12] |
| 6 | Osteosarcoma cells | COL2A1, ACAN, SOX9 | [4] |
| 7 | T cells | CCL5, CD69, GNLY | [13, 14] |
| 8 | Endothelial cells | CD34, PECAM1, GNG11 | [15, 16] |
| 9 | Pericytes | ACTA2, RGS5 | [17] |
| 10 | Macrophages | CD14, AIF1, CSF1R | [3, 9, 18] |
| 11 | B cells | JCHAIN, MZB1, TCL1A | [19, 20] |
| 12 | Macrophages | CD14, AIF1, CD74 | [2, 3, 18] |
| 13 | Myoblasts | MYOG, TNNT2, TTN | [21, 22] |

This table is generated from the public literature cited.

**Supplementary References**

1. Wu, M.F., et al., The M1/M2 spectrum and plasticity of malignant pleural effusion-macrophage in advanced lung cancer. Cancer Immunol Immunother, 2021. 70(5): p. 1435-1450.

2. Stables, M.J., et al., Transcriptomic analyses of murine resolution-phase macrophages. Blood, 2011. 118(26): p. e192-208.

3. Tsang, J.C.H., et al., Integrative single-cell and cell-free plasma RNA transcriptomics elucidates placental cellular dynamics. Proc Natl Acad Sci U S A, 2017. 114(37): p. E7786-e7795.

4. Baryawno, N., et al., A Cellular Taxonomy of the Bone Marrow Stroma in Homeostasis and Leukemia. Cell, 2019. 177(7): p. 1915-1932.e16.

5. Elsafadi, M., et al., Characterization of Cellular and Molecular Heterogeneity of Bone Marrow Stromal Cells. Stem Cells Int, 2016. 2016: p. 9378081.

6. Rowe, P.S., et al., MEPE has the properties of an osteoblastic phosphatonin and minhibin. Bone, 2004. 34(2): p. 303-19.

7. Twine, N.A., et al., Identification of differentiation-stage specific markers that define the ex vivo osteoblastic phenotype. Bone, 2014. 67: p. 23-32.

8. Mizoshiri, N., et al., Transduction of Oct6 or Oct9 gene concomitant with Myc family gene induced osteoblast-like phenotypic conversion in normal human fibroblasts. Biochem Biophys Res Commun, 2015. 467(4): p. 1110-6.

9. He, J., et al., Markers of adipose tissue macrophage content are negatively associated with serum HDL-C concentrations. Atherosclerosis, 2011. 215(1): p. 243-6.

10. Chen, H., et al., PU.1 (Spi-1) autoregulates its expression in myeloid cells. Oncogene, 1995. 11(8): p. 1549-60.

11. Lee, D., et al., HMGB2 is a novel adipogenic factor that regulates ectopic fat infiltration in skeletal muscles. Sci Rep, 2018. 8(1): p. 9601.

12. Dudakovic, A., et al., High-resolution molecular validation of self-renewal and spontaneous differentiation in clinical-grade adipose-tissue derived human mesenchymal stem cells. J Cell Biochem, 2014. 115(10): p. 1816-28.

13. Zheng, C., et al., Landscape of Infiltrating T Cells in Liver Cancer Revealed by Single-Cell Sequencing. Cell, 2017. 169(7): p. 1342-1356.e16.

14. Beura, L.K., et al., T Cells in Nonlymphoid Tissues Give Rise to Lymph-Node-Resident Memory T Cells. Immunity, 2018. 48(2): p. 327-338.e5.

15. Zhuo, J., W. Fu, and S. Liu, Correlation of contrast-enhanced ultrasound with two distinct types of blood vessels for the assessment of angiogenesis in lewis lung carcinoma. Ultraschall Med, 2014. 35(5): p. 468-72.

16. Han, X., et al., Mapping human pluripotent stem cell differentiation pathways using high throughput single-cell RNA-sequencing. Genome Biol, 2018. 19(1): p. 47.

17. Kim, N., et al., Single-cell RNA sequencing demonstrates the molecular and cellular reprogramming of metastatic lung adenocarcinoma. Nat Commun, 2020. 11(1): p. 2285.

18. Kováčiková, M., et al., Dietary intervention-induced weight loss decreases macrophage content in adipose tissue of obese women. Int J Obes (Lond), 2011. 35(1): p. 91-8.

19. Helmink, B.A., et al., B cells and tertiary lymphoid structures promote immunotherapy response. Nature, 2020. 577(7791): p. 549-555.

20. Brinas, F., R. Danger, and S. Brouard, TCL1A, B Cell Regulation and Tolerance in Renal Transplantation. Cells, 2021. 10(6).

21. Zeng, W., et al., Single-nucleus RNA-seq of differentiating human myoblasts reveals the extent of fate heterogeneity. Nucleic Acids Res, 2016. 44(21): p. e158.

22. Kong, X., et al., Establishment of myoblast cell line and identification of key genes regulating myoblast differentiation in a marine teleost, Sebastes schlegelii. Gene, 2021. 802: p. 145869.

**Table S2.** Primer sequences used in this study

| Gene | Forward | Reverse |
| --- | --- | --- |
| EFEMP2 | TGTCGAGAGCAGCCTTCA | CGGGGTAGACGGAGGTC |
| GALNT14 | CCCACCTCTGCCTCGATA | ATGGCCCTTGCTGCTTC |
